# High temperature inhibits photosynthesis of chrysanthemum (*Chrysanthemum morifolium* Ramat.) seedlings more than relative humidity

**DOI:** 10.3389/fpls.2023.1272013

**Published:** 2023-12-05

**Authors:** Jianfei Zhou, Xiaodong Jiang, Evgenios Agathokleous, Xiaojing Lu, Zaiqiang Yang, Ruiying Li

**Affiliations:** ^1^ Jiangsu Province Key Laboratory of Agricultural Meteorology, Nanjing, China; ^2^ Collaborative Innovation Center of Meteorological Disaster Forecasting and Assessment, Nanjing University of Information Science and Technology, Nanjing, China; ^3^ School of Applied Meteorology, Nanjing University of Information Science and Technology, Nanjing, China; ^4^ Meteorological Bureau of Heze City, Heze, China

**Keywords:** heat stress, humidity stress, chrysanthemum, photosynthesis, stomatal limitation, chlorophyll fluorescence

## Abstract

High relative humidity (RH) and high temperature are expected more frequently due to climate change, and can severely affect the growth of chrysanthemums. In order to analyze the interactive effects of RH and high temperature on the photosynthetic performance of chrysanthemum, a completely randomized block experiment was conducted with three factors, namely temperature (Day/night temperature, 35°C/18°C, 38°C/18°C, 41°C/18°C), RH (Whole day RH, 50%, 70%, 90%), and treatment duration (3d, 6d, 9d). The control (CK) temperature was 28°C/18°C and RH was 50%. The results showed that with the increase of temperature, the apparent quantum efficiency (AQE), maximum net photosynthetic rate (P_n-max_), net photosynthetic rate (P_n_), transpiration rate (T_r_), water use efficiency (WUE), maximal recorded fluorescence intensity (F_m_), PSII maximal photochemical efficiency (F_v_/F_m_), absorption flux per cross section (ABS/CSm), trapped energy flux per cross section (TRo/CSm), electron transport flux per cross section (ETo/CSm) and photosynthetic pigment content of leaves significantly decreased, the minimal recorded fluorescence intensity (F_o_), fluorescence intensity at point J of the OJIP curve (F_j_) and non-photochemical quenching per cross section (DIo/CSm) significantly increased, the fluorescence difference kinetics of the OJ phase of chrysanthemum leaves showed K-bands. P_n_, AQE, F_m_, F_v_, F_v_/F_m_, ABS/CSm, TRo/CSm, ETo/CSm and photosynthetic pigment content were higher at 70% RH than the other two RH conditions. The dominant factor causing the decrease of P_n_ in leaves was stomatal limitation at 35°C,38°C, three RH conditions, 3d and 6d, but non-stomatal limitation at 41°C and 9d. There was an interaction between temperature and RH, with a significant impact on P_n_. The temperature had the greatest impact on P_n_, followed by RH. This study confirms that heat stress severely affects the photosynthesis of chrysanthemum leaves, and when the temperature reaches or exceeds 35°C, adjusting the RH to 70% can effectively reduce the impact of heat stress on chrysanthemum photosynthesis.

## 1 Introduction

Chrysanthemum (*Chrysanthemum morifolium* Ramat.) is one of the four main cut flowers in the world (Fanourakis et al., 2022). It has been cultivated for more than 3,000 years, and over 3,000 varieties have been developed (Su et al., 2019). In China, it has a long history of use as a medicinal and edible plant (Ning et al., 2023). In Europe, the cultivation of chrysanthemum for cut flower production is a highly profitable industry (Castello et al., 2022). Chrysanthemum is one of the main flowers exported from China, with as many as 1.911 million chrysanthemums exported in 2019 alone (Dong, 2020). Hence, chrysanthemum is an economically important plant.

The optimal temperature for the growth of chrysanthemum is 25°C (Lee et al., 2013). Chrysanthemum growth is inhibited above 25°C and basically stops growing at 40°C (Li et al., 2021). Chrysanthemums ‘Shenma’ are typically short-day plants, and if the light duration is longer than 14.5 hours, its flowering will be delayed (Nakano et al., 2013). In order to meet the market supply demand and prompt chrysanthemums to bloom in the long sunshine season, black shading materials are often used for photoperiodic treatment, but the high temperature environment caused by shading in summer often causes chrysanthemums to wilt. Janka et al. (Janka et al., 2015) found that excessive irradiation and high temperatures above 28°C produced photoinhibition of chrysanthemum. The right relative humidity (RH) is equally important for the growth and development of chrysanthemum, and excessive RH can increase the incidence of white rust (Yoo and Roh, 2014). Influenced by the East Asian Monsoon, high temperature and high RH are main characteristics in greenhouse environment in southern China, which severely affect crops growth in glasshouses (Zhang et al., 2022). With global warming, extreme surface temperatures and duration in East Asia will increase more frequently (Chevuturi et al., 2018), and thus, the frequency of high temperature and high RH environments in greenhouses will be more frequent (Zheng et al., 2023). March to June is the main period for the growth of chrysanthemum seedlings in greenhouse in China and the impact of high temperature and high RH environment on the growth of greenhouse-grown chrysanthemum seedlings should be noticed. In previous studies, yield increase through moisture control has been demonstrated in strawberries (Zucchi et al., 2016) and roses (Mortensen and Gislerød, 2000), but not in chrysanthemum cultivation.

High temperature and RH affect various metabolic and physiological processes in plants. Photosynthetic parameters derived from the light response curve are important indicators of the light energy utilization capacity of plants in the study of adversity stress (Xu et al., 2022). Weng et al. (Weng et al., 2021) found that net photosynthetic rate (P_n_) of melon seedlings was inhibited at 42°C and 90% RH, compared to 30°C and 60% RH. In the study of stress, photosynthetic pigment content is an important index for assessing the extent of damage to photosynthetic organs, and chlorophylls are considered important components of stress biology in higher plants (Agathokleous et al., 2020). Yang et al. (Yang et al., 2022) found that lettuce chlorophyll a and chlorophyll b were reduced at 35°C and growth was inhibited, compared to 22°C. Chlorophyll fluorescence is inextricably linked to plant photosynthesis and can respond to changes in the photosynthetic system of a plant under adversity stress (Baker, 2008; Hazrati et al., 2016). Sun et al. (Sun et al., 2008) found that high temperatures reduced the PSII potential activity (F_v_/F_o_) and maximum photochemical efficiency (F_v_/F_m_) of chrysanthemum leaves, chrysanthemum protected reaction centers from damage by reducing the capture of light energy with the efficiency of electron transfer through PSII.

The two main reasons for limiting photosynthetic rate are stomatal and non-stomatal limitation. The exploration of stomatal and non-stomatal limitation under different adverse environments has been the focus of research, especially under combined stressors. Zubaidi et al. (Zubaidi et al., 2021) found that compared to 25°C, reduction in P_n_ in wheat at 32°C was not only due to lower stomatal conductance, but also non-stomatal effects as mesophyll conductance and quantum yield were lower. In addition, Barker et al. (Barker, 1990) found that increasing the RH of greenhouse significantly increased the stomatal conductance (G_s_), which improved the heat tolerance of tomato. While high temperatures have many detrimental impacts on plants, damage can be mitigated by regulating RH (Suzuki et al., 2015; Shamshiri et al., 2018). Zheng et al. (Zheng et al., 2020) found that increasing the RH to 70% at 35°C reduced gibberellin concentration (GA_3_) and increased abscisic concentration (ABA) in tomato shoots, which was favorable to the growth of tomato plants, compared to 28°C and 50% RH. Similarly, Xu et al. (Xu et al., 2020) found that high RH was effective in alleviating the limitation of tomato growth by high temperature and improving the root to crown ratio, compared to 50% RH. In rice, increasing RH by mist spray under heat stress increased chlorophyll content, P_n_ and yield (Jiang et al., 2020). When the effect of low, medium and high humidity on flowering and fruiting of tomato plants was studied (Peet et al., 2003), 50% RH was the optimum humidity at 35°C.

To our knowledge, there have been many studies on chrysanthemums under various factors of stress, but almost all of them are single-factor. The study of multifactorial stresses is more useful for chrysanthemum cultivation due to the complex environment in greenhouses. We hypothesized that high temperature could inhibit the photosynthesis of chrysanthemums, and changing RH could regulate it. The objective of this study is to analyze the interaction between RH and high temperature on the photosynthetic performance of chrysanthemums, analyze the dominant factors affecting chrysanthemum photosynthesis, and select the optimal RH for chrysanthemum photosynthesis in high temperature environments.

## 2 Materials and methods

### 2.1 Experimental design

The experiment was carried out in March 2022 at the Agricultural Meteorological Experiment Station of Nanjing University of Information Science and Technology (118°42′E, 32°12′N). Seedlings of *C. morifolium* cv. ‘Shenma’ were first raised in a greenhouse seedbed with substrate soil, vermiculite: peat mixture of 1:1 (v: v) in a Venlo-type glass greenhouse. When the seedlings had four true leaves, they were planted into pots of 17.5 cm (height) × 15.0 cm (diameter). The contents of organic carbon, available phosphorus, available potassium and nitrogen of the soil substrate were 10,450, 32.8, 89.4 and 1540 mg·kg^-1^. Soil texture was medium loam. Soil pH was 6.7. One plant was planted in each pot. Two weeks after planting, the plants were moved into artificial climate chambers (TGP-1260, Australia) in a laboratory.

The experiment was conducted to simulate a summer greenhouse by setting the environmental parameters of artificial climate chambers. There were three temperature (day/night temperature) conditions set as 35°C/18°C, 38°C/18°C and 41°C/18°C, and three RH conditions, namely 50%, 70% and 90%, in combination with each temperature condition. Moreover, the temperature treatment was conducted for 3, 6 and 9 d to study the time dependency of response to temperature and RH. The experiment was a completely randomized group design with a total of 27 treatment combinations (**Table 1**). The control (CK) temperature and RH were 28°C/18°C and 50% respectively, which are the optimum temperature and normal RH for chrysanthemums cultivation in Nanjing. The environmental conditions and management measures in the artificial climate chambers remained the same during the experiment, except for different settings of temperature and relative air humidity. The photoperiod (day/night time) was set to 7:00 a.m. - 17:00 p.m./18:00 p.m. - 6:00 a.m.The light intensity in the artificial climate chamber was 800 μmol·m^-2^·s^-1^ during daytime and 0 μmol·m^-2^·s^-1^ during nighttime.

**Table 1:** Experimental scheme of artificial climate chambers.

Treatment	Day/night temperature	RH	Treatment duration
	(°C)	(%)	(d)
T_1_	35/18	50	3
T_2_	35/18	50	6
T_3_	35/18	50	9
T_4_	35/18	70	3
T_5_	35/18	70	6
T_6_	35/18	70	9
T_7_	35/18	90	3
T_8_	35/18	90	6
T_9_	35/18	90	9
T_10_	38/18	50	3
T_11_	38/18	50	6
T_12_	38/18	50	9
T_13_	38/18	70	3
T_14_	38/18	70	6
T_15_	38/18	70	9
T_16_	38/18	90	3
T_17_	38/18	90	6
T_18_	38/18	90	9
T_19_	41/18	50	3
T_20_	41/18	50	6
T_21_	41/18	50	9
T_22_	41/18	70	3
T_23_	41/18	70	6
T_24_	41/18	70	9
T_25_	41/18	90	3
T_26_	41/18	90	6
T_27_	41/18	90	9

RH represents relative humidity.

### 2.2 Measurement

The light response curve, gas exchange parameters, photosynthetic pigment content and chlorophyll fluorescence of chrysanthemum leaves were measured at 4, 7 and 10 d after the start of the artificial control experiment.

#### 2.2.1 Light response curves

With three LI-6400 portable gas exchange analyzers (LI-COR Biosciences Inc, USA), the light response curves of leaves were recorded from 9:00 a.m.-11:30 a.m. on each observation day. Inside the leaf chamber, the CO_2_ level was maintained at 400 μmol·mol^-1^. The levels of photosynthetically active radiation (PAR) (μmol·m^-2^·s^-1^) inside the leaf chamber were 1200, 1000, 800, 600, 400, 300, 200, 100, and 0. The maximum wait time after each change of light intensity was set to 180s and the minimum wait time was 120s. Photosynthetic parameters like apparent quantum efficiency (AQE), light saturation point (LSP), maximum net photosynthetic rate (P_n-max_), light compensation point (LCP) and dark respiration rate (R_d_) were estimated using photosynthetic model simulations of Ye (Ye, 2010). The third leaf that was fully expanded before the experiment began was measured by the light response curve. One leaf per plant and three plants per treatment were measured.


(1) $$P_{n}=\frac{\alpha I+P_{n-max}-\sqrt{{\left(\alpha I+P_{n-max}\right)}^{2}-4\theta \alpha IP_{n-max}}}{2\theta }-R_{d}$$

Where, *P_n_* (μmol·m^-2^·s^-1^) means net photosynthetic rate. *θ* means curvature of the curve. *I* (μmol·m^-2^·s^-1^) means light intensity. *R_d_* (μmol·m^-2^·s^-1^) means dark respiration rate. α means initial quantum efficiency. *P_n-max_* (μmol·m^-2^·s^-1^) means maximum net photosynthetic rate.

#### 2.2.2 Gas exchange parameters

Gas exchange parameters of chrysanthemum leaves were measured using the transparent leaf chamber that comes with the LI-6400 portable gas exchange analyzer (LI-COR Biosciences Inc, USA). Measurement time, leaf position was the same as described in section 2.2.1. The net photosynthetic rate (P_n_), stomatal conductance (G_s_), intercellular CO_2_ concentration (C_i_), atmospheric CO_2_ concentration (C_a_) and transpiration rate (T_r_) were measured. Vapor pressure deficit (VPD) was calculated as formula 2 (Howell and Dusek, 1995; Kadam et al., 2015).The stomatal restriction values (L_s_) were calculated as formula 3 (Berry, 1982). The water use efficiency (WUE) was calculated as formula 4 (R A Fischer and Turner, 1978).


(2) $$VPD=0.611e^{\frac{17.27\times T}{T+237.3}}\times (1-RH)$$

(3) $$Ls=1-C_{i}/C_{a}$$

(4) $$WUE=\frac{P_{n}}{T_{r}}$$

#### 2.2.3 Photosynthetic pigment content

The photosynthetic pigment content of the leaves was measured according to the method of Li (Li, 2000). A randomly derived amount of 0.2g of leaf tissue was placed into 25ml of 95% ethanol left in dark at room temperature for 48h until the photosynthetic pigments in the leaves were completely leached out. The absorbance was measured colorimetrically at 665, 649 and 470 nm using a UV-1800 instrument (SHIMADZU, Japan). The photosynthetic pigment concentration (mg·g^-1^) was calculated using formulas 5-9.


(5) $$Chla=13.95D_{665}-6.88D_{649}$$

(6) $$Chlb=24.96D_{649}-7.32D_{665}$$

(7) Chl=Chla+Chlb

(8) $$Car=(1000D_{470}-2.05Chla-114.8Chlb)/245$$

(9) $$\begin{array}{c} Pigment\;content(mg\cdot g^{-1})\\ =pigment\;concentration\\ \times volume\;of\;extraction\;solution/fresh\;mass\;of\;sample \end{array}$$

Where, *Chla* (mg·g^-1^), *Chlb* (mg·g^-1^), *Car* (mg·g^-1^) and *Chl* (mg·g^-1^) mean the concentrations of chlorophyll a, chlorophyll b, carotenoids and chlorophyll (a+b), respectively. *D_665_*, *D_649_*, and *D_470_* mean the absorbance of the extracts at 665, 649, and 470 nm, respectively.

#### 2.2.4 Chlorophyll fluorescence parameters

Chlorophyll fluorescence induction curves can provide insight into know the response of the plant to changes in environmental factors. Therefore, here we measured the chlorophyll fluorescence of each treatment. Chlorophyll fluorescence parameters were measured by a plant efficiency analyzer (Pocket PEA, Hansatech, UK). Measurement time and blade position were the same as the photosynthetic parameters. Leaves were dark-adapted with leaf clips for 30 minutes before measurement. Following a 5000 μmol·m^-2^·s^-1^ light pulse, the fast chlorophyll fluorescence-induced kinetic curve (OJIP) and its related fluorescence parameters of chrysanthemum leaves were measured with a duration of 1s. The minimal recorded fluorescence intensity (F_o_), fluorescence intensity at point J of the OJIP curve (F_j_), maximal recorded fluorescence intensity (F_m_), PSII maximal photochemical efficiency (F_v_/F_m_) were measured. The JIP-test indices and terminology mentioned in this paper are presented in **Table 2** (Strasser et al., 2010). The variable fluorescence intensities were O-J normalized according to formula 10-11 (Yusuf et al., 2010).

**Table 2:** JIP-test indices and terminology used in the study.

Terms and Formulas	Illustrations
ABS/CSm ≈ F_m_	absorption flux per cross section
TRo/CSm = (1- F_o_/F_m_)·(ABS/CSm)	trapped energy flux per cross section
ETo/CSm = (1-F_o_/F_m_)·(1- (F_J_-F_o_)/(F_m_-F_o_)·(ABS/CSm)	electron transport flux per cross section
DIo/CSm = (ABS/CSm) - (TRo/CSm)	non-photochemical quenching per cross section


(10) $$W_{oj}=(F_{t}-F_{o})/(F_{j}-F_{o})$$

(11) $$\Delta W=W_{oj}-W_{ck}$$

Where, *W_oj_* means fluorescence difference kinetics. *F_t_* means instantaneous fluorescence at any moment. *F_o_* means minimal recorded fluorescence intensity. *F_j_* means fluorescence intensity at point J of the OJIP curve.

### 2.3 Data processing

All values were the means of three replicates per treatment. In order to make comparisons across treatments with different temperature, RH and treatment duration, this study used the calculation of mean values of relevant treatment indicators for comparison. The values of T_35°C_, T_38°C_ and T_41°C_ were the means of treatments which day/night temperature was set as 35°C/18°C, 38°C/18°C and 41°C/18°C, respectively. The values of RH_50%_, RH_70%_ and RH_90%_ were the means of treatments which RH condition was set as 50%, 70% and 90%, respectively. The values of L_3d_, L_6d_ and L_9d_ were the means of treatments which duration was set as 3d, 6d and 9d, respectively. Duncan’s multiple range test at the 0.05 level of significance was used to detect differences between all treatments using SPSS 26.0 software (SPSS Inc., Chicago, IL). Figures were drawn using Origin Pro 2023b (Origin Lab Corporation, Northampton, MA, USA).

## 19 (10), 2149-21543 Results

### 3.1 Interactive effects of relative humidity and high temperature on light response curves

**Table 3** shows the parameters of light response curves of chrysanthemum leaves under different treatments. As to temperature, the values of LSP, AQE and P_n-max_ in CK treatment were significantly higher than that of all temperature treatments. AQE and P_n-max_ values decreased with increasing temperature. The values of LSP, AQE, and P_n-max_ in T_41°C_ were the lowest among all temperature treatments, which were 36.67%, 47.79% and 57.61% lower than that of CK treatment. The values of LCP and R_d_ in three temperature treatments were higher than that of CK treatment.

**Table 3:** Parameters of light response curve of chrysanthemum leaves.

Treatments	LSP	LCP	AQE	P_n-max_	R_d_
	(μmol·m^-2^·s^-1^)	(μmol·m^-2^·s^-1^)		(μmol·m^-2^·s^-1^)	(μmol·m^-2^·s^-1^)
CK	1132 ± 36a	14 ± 1c	0.113 ± 0.0011a	13.54 ± 0.63a	0.63 ± 0.020d
T_35°C_	809 ± 36b	35 ± 8b	0.088 ± 0.010b	9.73 ± 1.30b	0.94 ± 0.14c
T_38°C_	788 ± 34bc	42 ± 8ab	0.077 ± 0.011bc	7.74 ± 1.27cd	1.18 ± 0.14ab
T_41°C_	717 ± 34d	50 ± 7ab	0.059 ± 0.013e	5.74 ± 0.97e	1.30 ± 0.14a
RH_50%_	763 ± 44bcd	39 ± 8b	0.077 ± 0.014d	7.58 ± 1.92cde	1.16 ± 0.19abc
RH_70%_	807 ± 47b	38 ± 9b	0.080 ± 0.015bc	8.88 ± 1.97bc	1.03 ± 0.18bc
RH_90%_	743 ± 44cd	50 ± 8a	0.067 ± 0.018d	6.76 ± 1.75de	1.24 ± 0.18a
L_3d_	797 ± 48b	38 ± 9b	0.080 ± 0.014bc	8.53 ± 2.11bcd	1.03 ± 0.19bc
L_6d_	773 ± 47bc	41 ± 9ab	0.075 ± 0.016bcd	7.79 ± 2.04cd	1.12 ± 0.18abc
L_9d_	743 ± 47cd	49 ± 9a	0.068 ± 0.017cd	6.89 ± 1.76de	1.27 ± 0.18a
T	**	**	**	**	**
RH	**	**	**	**	**
L	**	**	/	/	**
T×RH	*	/	/	**	/
T×L	*	/	*	**	/
RH×L	/	*	**	**	/
T×RH×L	/	/	*	*	/

Values are the means of three replicates per treatment. T, RH and L represent temperature, relative humidity and treatment duration, respectively. ± indicates standard deviation. Means are not significantly different between different treatments when followed by the same lowercase letter, means are significantly different between different treatments (P < 0.05) when followed by different lowercase letters. * and * * indicate P < 0.05 and P < 0.01. LSP means light saturation point. LCP means light compensation point. AQE means apparent quantum efficiency. P_n-max_ means maximum net photosynthetic rate. R_d_ means dark respiration rate.

Among RH treatments, the values of AQE in RH_70%_ were highest and significantly larger than that of other RH treatments. Although statistically non-significant, RH_70%_ had the highest values of LSP and P_n-max_, followed by the RH_50%_ and RH_90%_. The value of R_d_ in RH_70%_ was lower than that of RH_90%_, indicating that the respiratory consumption of leaves was low and photosynthetic activity was high at 70% RH, while the respiratory consumption of leaves was high at 90% RH.

Regarding the treatment duration, although statistically non-significant, the values of LSP, AQE, and P_n-max_ decreased over time. The values of LSP, AQE, and P_n-max_ in L_9d_ were the lowest among L_3d_, L_6d_ and L_9d_, which were 34.82%, 39.82% and 49.11% lower than that of CK treatment.

**Figure 1:**
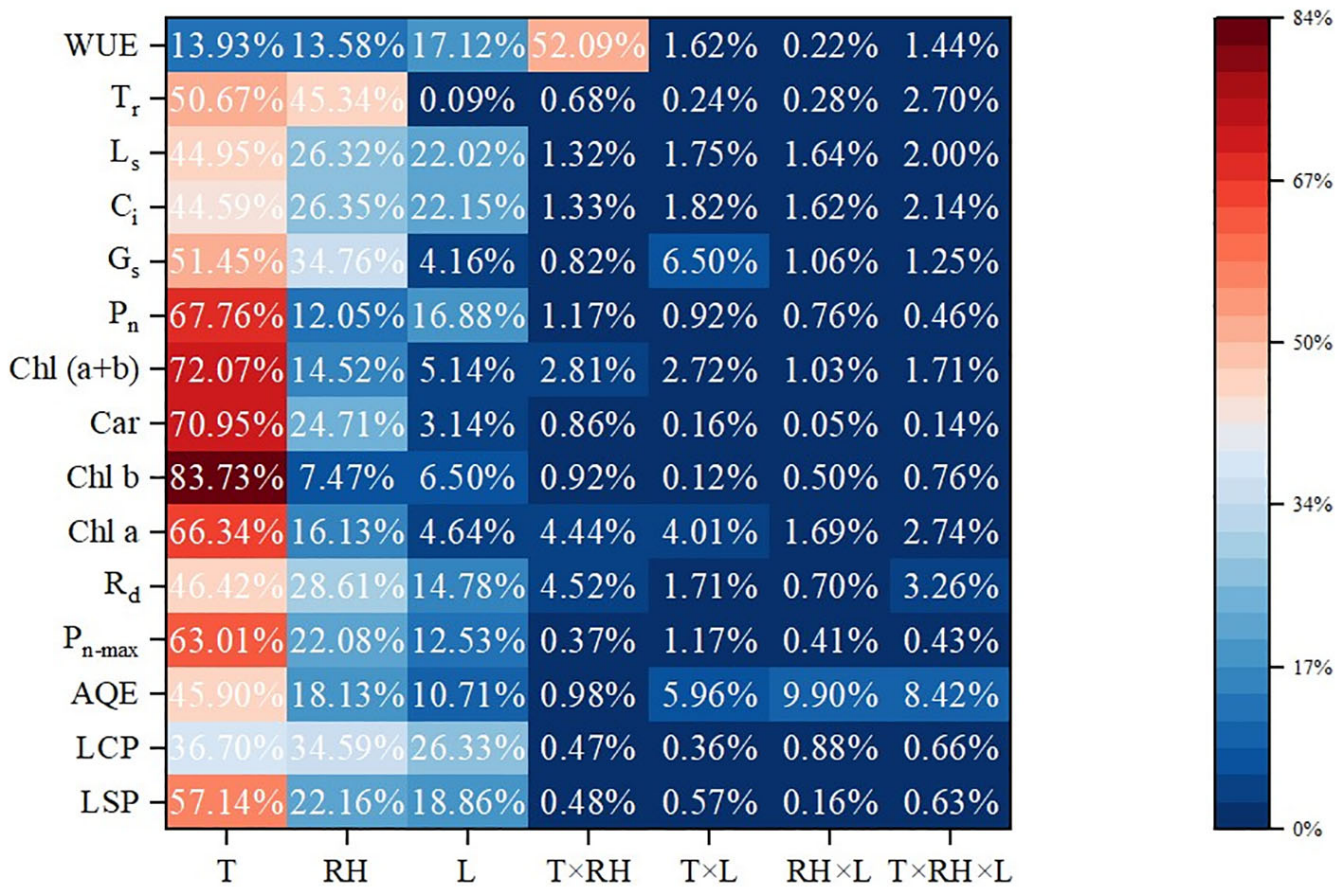
Contributions of temperature, RH and treatment duration. Contribution rate (%) =SS_F_×100/(SS_T_-SS_E_-SS_B_). SS_F_ means sum of squares away from the mean difference. SS_T_, SS_E_ and SS_B_ mean sum of squares for total, error and block, respectively. T, RH and L represent temperature, relative humidity and treatment duration, respectively.

The results of ANOVA are also shown in **Table 3**. Temperature and RH had a significant (P< 0.01) impact on light response curve parameters. Treatment duration had a significant (P< 0.01) impact on LCP, LSP and R_d_. The interaction between temperature and RH was significant for P_n-max_ (P< 0.01) and LSP (P< 0.05). Besides, the interaction between temperature and treatment duration had a significant impact on P_n-max_ (P< 0.01), LSP and AQE (P< 0.05). Moreover, the interaction between RH and treatment duration had a significant impact on AQE and P_n-max_ (P< 0.01) and LCP (P< 0.05). Further, the interaction of all three factors (temperature, RH and treatment duration) was significant for AQE and P_n-max_ (P< 0.05). Contributions of temperature, RH and treatment duration are shown in **Figure 1**. Temperature had the most impact, since it contributed 57.14%, 36.70%, 45.89%, 63.01% and 46. 42% of the variation in LSP, AQE, P_n-max_, LCP and R_d_, followed by RH, and treatment duration was the least.

**Figure 2:**
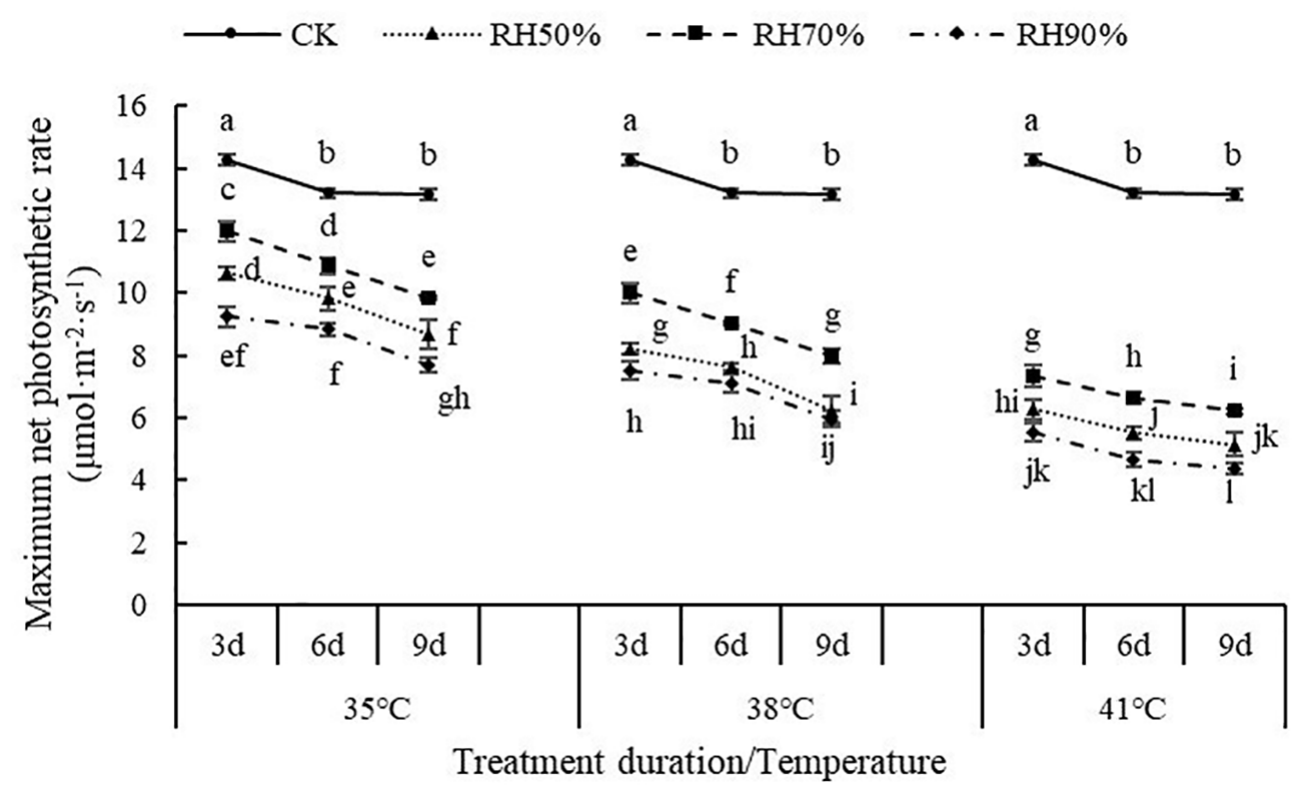
Effect of interaction between temperature and RH on maximum net photosynthetic rate of chrysanthemum leaves.

The interaction between temperature and RH was significant for P_n-max_ (P< 0.01). Interactions affecting P_n-max_ from looking at temperature effects at a given RH and RH effects at a given temperature were shown in **Figure 2**. At a given temperature, the values of P_n-max_ at 70% RH were highest, followed by 50% RH and 90% RH, which indicated that 70% RH mitigated the negative effect of high temperature on P_n-max_, while high RH aggravated. At a given RH, the values of P_n-max_ decreased with increasing temperature, which indicated that increasing temperature aggravated the effect of RH on P_n-max_. In addition, the values of P_n-max_ decreased with increasing treatment duration. The value of P_n-max_ was lowest at 41°C and 90% RH.

### 3.2 Interactive effects of relative humidity and high temperature on photosynthetic pigment content

**Figure 3:**
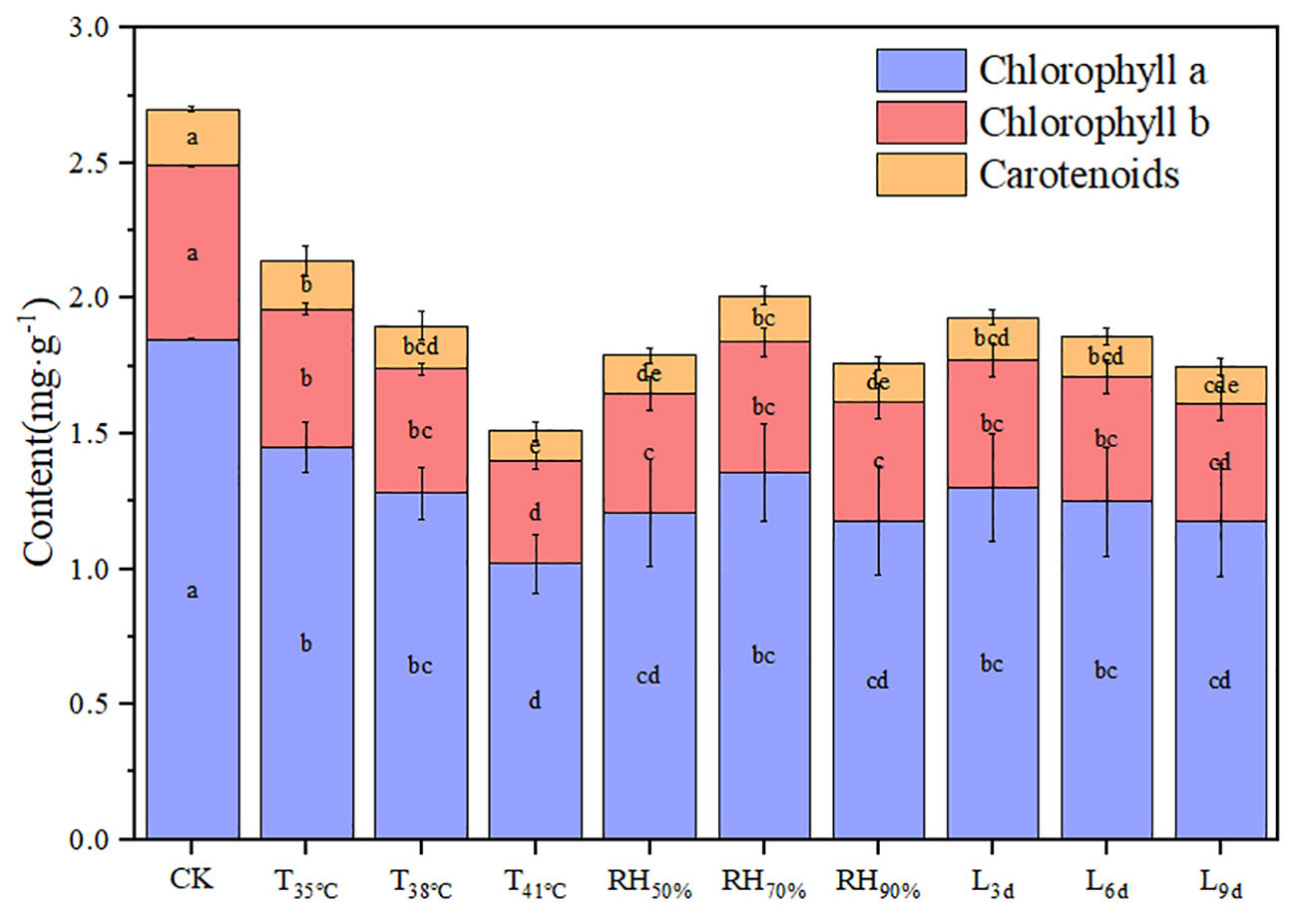
Effect of different temperature, RH and treatment duration on photosynthetic pigment content of chrysanthemum leaves.

**Figure 3** shows the photosynthetic pigment contents of chrysanthemum leaves under different treatments. The values of chlorophyll a, chlorophyll b, carotenoids, and chlorophyll (a+b) generally decreased with increasing temperature. The values of chlorophyll a, chlorophyll b, carotenoids, and chlorophyll (a+b) in T_41°C_ were the lowest among three temperature treatments, which were 44.86%, 40.63%, 47.62% and 43.78% lower than that of CK treatment.

The value of carotenoids in RH_70%_ was highest and significantly higher than that of other RH treatments. Although without statistically significant, the values of chlorophyll a, chlorophyll b, and chlorophyll (a+b) decreased in RH_50%_ and RH_90%_, compared to RH_70%_. There was no significant effect of treatment duration on photosynthetic pigment content.

Temperature had the most contribution to the variation in the values of chlorophyll a, chlorophyll b, carotenoids and chlorophyll (a+b), which was 66.34%, 83.731%, 70.95% and 72.07%, followed by RH, and treatment duration was the least.

**Figure 4:**
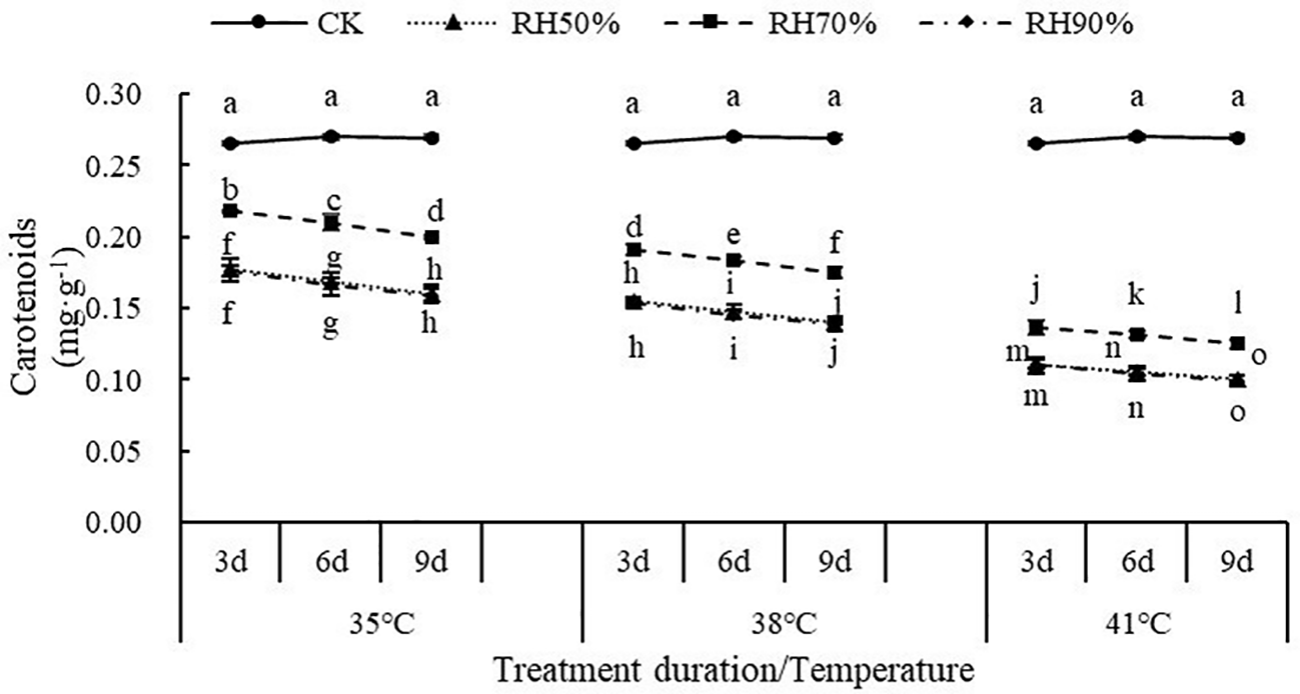
Effect of interaction between temperature and RH on carotenoids of chrysanthemum leaves.

The interaction between temperature and RH was significant for carotenoids (P< 0.01). Interactions affecting carotenoids from looking at temperature effects at a given RH and RH effects at a given temperature were shown in **Figure 4**. At a given temperature, the values of carotenoids at 70% RH were highest, which indicated that 70% RH mitigated the negative effect of high temperature on carotenoids. The values of carotenoids at 50% RH and 90% RH were significantly lower than that of CK, which indicated that 50% RH and 90% RH aggravated the negative effect of high temperature on carotenoids. At a given RH, the values of carotenoids decreased with increasing temperature, which indicated that increasing temperature aggravated the effect of RH on carotenoids. In addition, the values of carotenoids decreased with increasing treatment duration.

### 3.3 Interactive effects of relative humidity and high temperature on gas exchange parameters

**Table 4** shows the gas exchange parameters of chrysanthemum leaves under different treatments. The value of P_n_ in CK treatment was significantly higher than that of all treatments. With increasing temperature, the value of P_n_ significantly decreased and to reach the lowest in T_41°C_, which was 57.61% lower than that of CK. Among RH treatments, although statistically non-significant, the value of P_n_ was highest in RH_70%_, followed by RH_50%_, and RH_90%_ was the lowest. There was no significant effect of treatment duration on P_n_.

**Table 4:** Gas exchange parameters of chrysanthemum leaves.

Treatments	VPD	P_n_	G_s_	C_i_	L_s_	T_r_	WUE
	kPa	(μmol·m^-2^·s^-1^)	(mol·m^-2^·s^-1^)	(μmol·mol^-1^)		(mmol·m^-2^·s^-1^)	(%)
CK	1.89	13.14 ± 0.61a	0.25 ± 0.0059a	305 ± 4a	0.24 ± 0.0096d	3.26 ± 0.015a	4.02 ± 0.17a
T_35°C_	1.69	9.44 ± 1.26b	0.19 ± 0.022bc	245 ± 21b	0.39 ± 0.060c	3.05 ± 0.081b	3.09 ± 0.35b
T_38°C_	1.99	7.51 ± 1.23cd	0.14 ± 0.017bc	187 ± 20d	0.53 ± 0.056a	2.86 ± 0.083e	2.62 ± 0.38bc
T_41°C_	2.33	5.57 ± 0.94e	0.17 ± 0.018d	228 ± 21bc	0.43 ± 0.059bc	2.91 ± 0.079bc	1.81 ± 0.28d
RH_50%_	3.34	7.35 ± 1.87cde	0.17 ± 0.022c	222 ± 26bc	0.44 ± 0.063bc	2.95 ± 0.095b	2.49 ± 0.60bc
RH_70%_	2.00	8.61 ± 1.91bc	0.19 ± 0.023b	242 ± 27b	0.40 ± 0.065c	3.05 ± 0.078b	2.82 ± 0.60bc
RH_90%_	0.67	6.56 ± 1.70de	0.15 ± 0.020d	196 ± 26cd	0.51 ± 0.065ab	2.87 ± 0.082de	2.28 ± 0.57cd
L_3d_	/	8.28 ± 2.04bcd	0.17 ± 0.028cd	225 ± 35b	0.44 ± 0.090c	3.05 ± 0.081b	2.71 ± 0.65bc
L_6d_	/	7.56 ± 1.97cd	0.16 ± 0.028bc	215 ± 33bc	0.46 ± 0.086bc	2.86 ± 0.083e	2.64 ± 0.68bc
L_9d_	/	6.69 ± 1.71de	0.17 ± 0.026bc	220 ± 31cd	0.45 ± 0.078ab	2.97 ± 0.079bc	2.25 ± 0.56cd
T	/	**	**	**	**	**	*
RH	/	**	**	**	**	**	/
L	/	/	/	**	**	**	/
T×RH	/	**		/	/	/	**
T×L	/	*	**	/	/	/	/
RH×L	/	/	/	/	/	/	/
T×RH×L	/	/	/	/	/	*	/

Values are the means of three replicates per treatment. T, RH and L represent temperature, relative humidity and treatment duration, respectively. ± indicates standard deviation. Means are not significantly different between different treatments when followed by the same lowercase letter, means are significantly different between different treatments (P < 0.05) when followed by different lowercase letters. * and * * indicate P < 0.05 and P < 0.01. LSP means light saturation point. VPD means the vapor pressure deficit. P_n_ means net photosynthetic rate. G_s_ means stomatal conductance. C_i_ means intercellular CO_2_ concentration. L_s_ means stomatal restriction values. T_r_ means transpiration rate. WUE means water use efficiency.

Compared to CK treatment, the values of G_s_ and C_i_ in all treatments significantly decreased. The lowest values of G_s_ and C_i_ were recorded in T_38°C_, which were 52.00% and 42.40% lower than that of CK treatment. Among RH treatments, the value of G_s_ was highest in RH_70%_, followed by RH_50%_, and RH_90%_ was the lowest. The value of C_i_ in RH_70%_ was higher than that of RH_90%_. The values of G_s_ and C_i_ in L_6d_ were the lowest among L_3d_, L_6d_ and L_9d_, which were 48.00% and 38.66% lower than that of CK treatment.

The value of L_s_ in CK treatment was significantly lower than that of all treatments. Among temperature treatments, the value of L_s_ was highest in T_38°C_, which was 79.17% higher than that of CK treatment, followed by T_41°C_, and T_35°C_ was the lowest. The value of L_s_ in RH_70%_ was lower than that of RH_50%_ and RH_90%_. There was no significant effect of treatment duration on L_s_.

In order to determine whether the decrease in P_n_ is caused by obstruction of CO_2_ diffusion or decrease in enzyme activity, we conducted stomatal and non-stomatal limitation analyses of photosynthesis. The evaluation of stomatal and non-stomatal limitation mainly depends on the change direction of C_i_ and L_s_ (Xu, 1997).The increase of L_s_ and the decrease of C_i_ indicate that the main reason for P_n_ decrease is stomatal limitation, while the decrease of L_s_ and the increase of C_i_ indicate the reason is non-stomatal. Compared to CK, L_s_ increased and C_i_ decreased in T_35°C_, indicating that stomatal limitation was the reason for P_n_ decrease. In addition, the reason for T_38°C_ was also stomatal limitation because L_s_ increased and C_i_ decreased, compared to T_35°C_. In T_41°C_, L_s_ decreased while C_i_ increased, compared to T_38°C_, indicating that the non-stomatal limitation was the reason for P_n_ decrease. Under three RH conditions, compared to CK, L_s_ increased and C_i_ decreased, indicating that RH affects P_n_ through stomatal factors. L_s_ increased and C_i_ decreased under L_3d_ and L_6d_ treatments, indicating that the major factor for P_n_ decrease from 0d to 6d was stomatal limitation. Compared to L_6d_, L_s_ decreased and C_i_ increased in L_9d_ treatment, indicating that the decrease in P_n_ from 6d to 9d was non-stomatal limitation.

For the water regimes of chrysanthemums, the values of T_r_ and WUE in CK treatment were significantly higher than that of all treatments. Among temperature treatments, the value of T_r_ was highest in T_35°C_, followed by T_41°C_, and T_38°C_ was the lowest, which was 12.07% lower than that of CK treatment. With increasing temperature, the value of WUE significantly decreased and reached the lowest in T_41°C_, which was 54.98% lower than that of CK treatment. The value of T_r_ in RH_50%_ and RH_70%_ was significantly higher than that of RH_90%_. Although statistically non-significant, the value of WUE was highest in RH_70%_, followed by RH_50%_, and RH_90%_ was the lowest. The value of T_r_ was highest in L_3d_, followed by L_9d_, and L_6d_ was the lowest, which was 12.07% lower than that of CK treatment. There was no significant effect of treatment duration on WUE.

The results of ANOVA are also shown in **Table 4**. Temperature and RH had significant impacts (P<0.01) on P_n_, G_s_, C_i_ and L_s_. Temperature and treatment duration had significant impacts (P<0.01) on T_r_. The interaction of temperature and RH was significant for P_n_ and WUE (P< 0.01). Furthermore, the interaction of temperature and treatment duration was significant for G_s_ and P_n_ (P< 0.05). Temperature had the greatest impacts on P_n_, G_s_, C_i_, L_s_ and T_r_ with contribution rates of 67.76%, 51.45%, 44.59%, 44.95% and 50.67%, followed by RH. The interaction of temperature and RH had the greatest impact on WUE with contribution rates of 52.09%.

**Figure 5:**
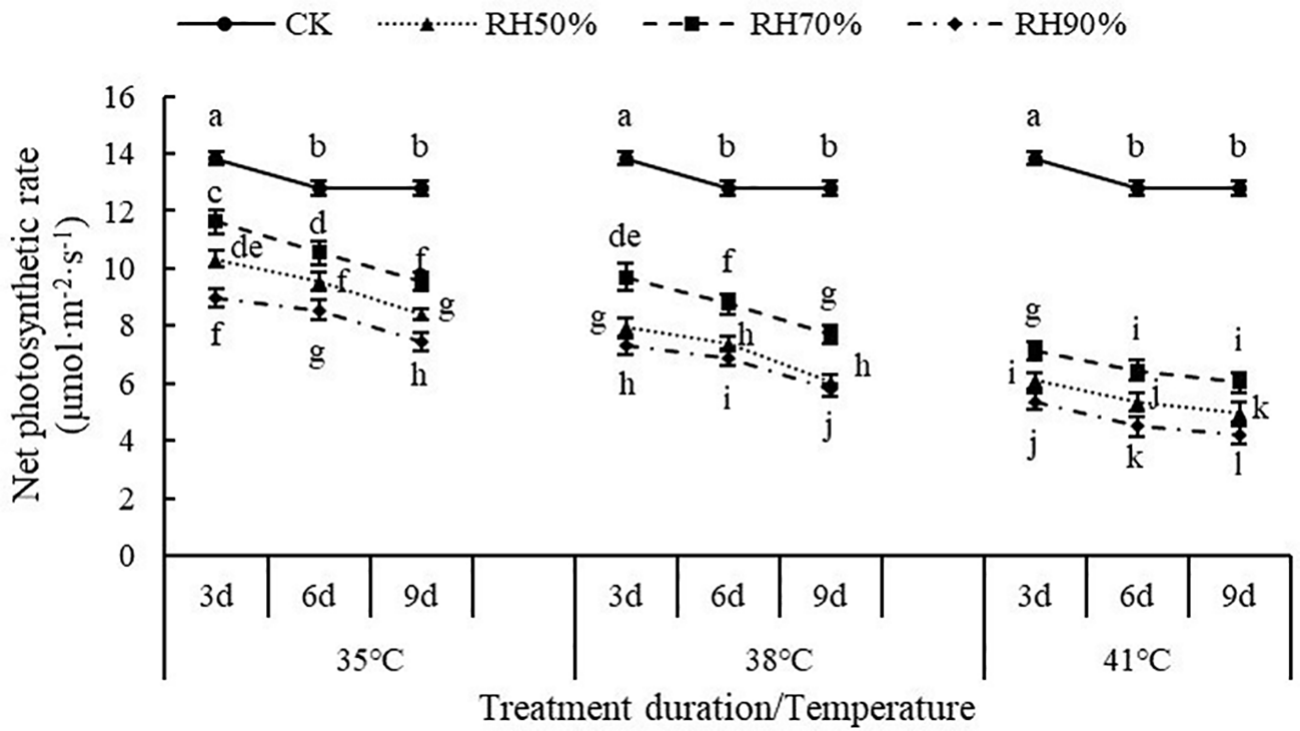
Effect of interaction between temperature and RH on net photosynthetic rate of chrysanthemum leaves.

The interaction between temperature and RH was significant for P_n_ (P< 0.01). Interactions affecting P_n_ from looking at temperature effects at a given RH and RH effects at a given temperature were shown in **Figure 5**. At a given RH, the values of P_n_ decreased with increasing temperature, which indicated that increasing temperature aggravated the effect of RH on P_n_. At a given temperature, the values of P_n_ at 70% RH were highest, followed by 50% RH and 90% RH, which indicated that 70% RH mitigated the negative effect of high temperature on P_n_, while high RH aggravated. In addition, the values of P_n_ decreased with increasing treatment duration. The value of P_n_ was lowest at 41°C and 90% RH.

### 3.4 Interactive effects of relative humidity and high temperature on chlorophyll fluorescence

**Figure 6:**
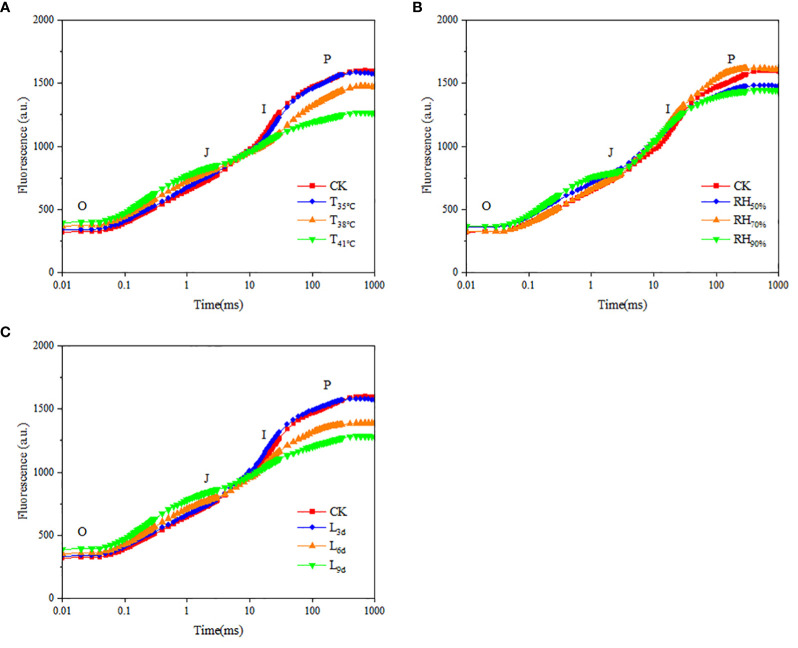
Effect of different temperature **(A)**, RH **(B)** and treatment duration **(C)** on rapid fluorescence kinetic curve of chrysanthemum leaves. T, RH and L represent temperature, relative humidity and treatment duration, respectively.

Chlorophyll fluorescence kinetic curves respond to the photosynthetic efficiency and potential of chrysanthemum leaves. As seen in **Figure 6A**, the fluorescence values of curve O-P in CK treatment, T_35°C_, T_38°C_ and T_41°C_ were 318~1595, 336~1547, 367~1470 and 393~1264, indicating that the fluorescence values of the leaves decreased with increasing temperature. As seen in **Figure 6B**, the fluorescence values of curve O-P in RH_50%_, RH_70%_ and RH_90%_ were 356~1475, 322~1604 and 363~1441. It showed that the fluorescence value of curve O-P in RH_70%_ was highest, followed by RH_50%_, and RH_90%_ was the lowest. As seen in **Figure 6C**, the fluorescence values of curve O-P in L_3d_, L_6d_ and L_9d_ were 332~1570, 355~1385 and 389~1282, indicating that the fluorescence values of chrysanthemum leaves decreased with increasing treatment duration.

As seen in **Table 5**, F_o_ and F_j_ were lower in CK treatment than that of all temperature treatments. With temperature increasing, F_o_ and F_j_ increased and reached the highest in T_41°C_, which were 23.53% and 13.93% higher than that of CK treatment. F_o_ and F_j_ in RH_70%_ were close to that of CK treatment, while lower than that of other RH treatments. With treatment duration increasing, F_o_ and F_j_ increased and reached the highest in L_9d_, which were 22.29% and 15.59% higher than that of CK treatment. F_m_ and F_v_/F_m_ were higher in CK than that of all temperature treatments. With temperature increasing, F_m_ and F_v_/F_m_ decreased and reached the lowest in T_41°C_, which were 20.69% and 13.75% lower than that of CK treatment. F_m_ and F_v_/F_m_ in RH_70%_ were close to that of CK treatment, while higher than that of other RH treatments. With treatment duration increasing, F_m_ and F_v_/F_m_ decreased and reached the lowest in L_9d_, which were 19.56% and 13.75% lower than that of CK treatment.

**Table 5:** Chlorophyll fluorescence parameters of chrysanthemum leaves.

Treatments	F_o_	F_j_	F_m_	F_v_/F_m_
CK	323 ± 3d	725 ± 6d	1600 ± 14a	0.8 ± 0.002a
T_35°C_	341 ± 4c	750 ± 6c	1583 ± 20b	0.78 ± 0.002b
T_38°C_	373 ± 3b	789 ± 7b	1475 ± 22c	0.75 ± 0.003c
T_41°C_	399 ± 5a	826 ± 7a	1269 ± 18e	0.69 ± 0.002d
RH_50%_	362 ± 4b	772 ± 6b	1483 ± 26c	0.76 ± 0.002c
RH_70%_	326 ± 3d	730 ± 6d	1618 ± 23a	0.79 ± 0.003ab
RH_90%_	369 ± 4b	788 ± 7b	1449 ± 20c	0.75 ± 0.002c
L_3d_	337 ± 3c	729 ± 6c	1581 ± 22b	0.79 ± 0.002ab
L_6d_	360 ± 4b	767 ± 7b	1388 ± 24d	0.74 ± 0.003c
L_9d_	395 ± 3a	838 ± 6a	1287 ± 23e	0.69 ± 0.003d

Values are the means of three replicates per treatment. T, RH and L represent temperature, relative humidity and treatment duration, respectively. ± indicates standard deviation. Means are not significantly different between different treatments when followed by the same lowercase letter, means are significantly different between different treatments (P < 0.05) when followed by different lowercase letters. LSP means light saturation point. F_o_ means minimal recorded fluorescence intensity. F_j_ means fluorescence intensity at point J of the OJIP curve. F_m_ means maximal recorded fluorescence intensity. F_v_/F_m_ means PSII maximal photochemical efficiency.

**Figure 7:**
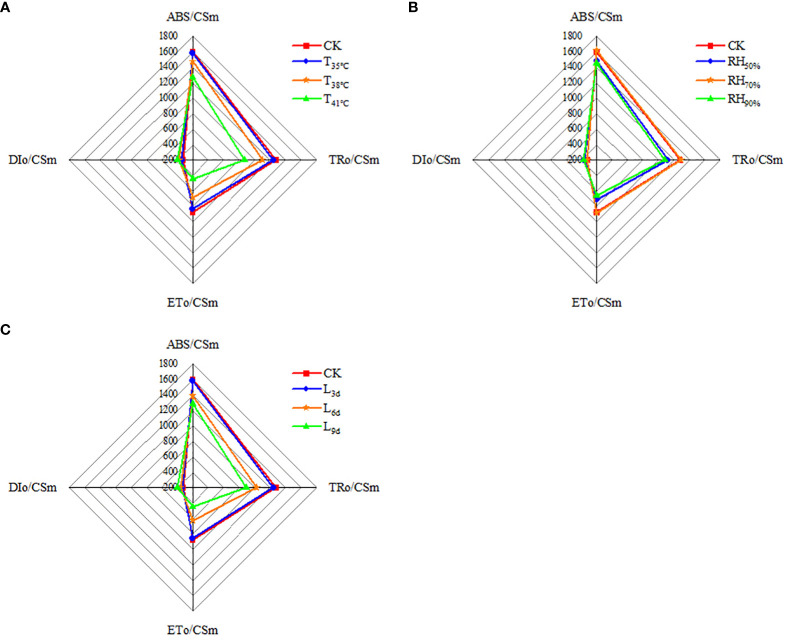
Effect of different temperature **(A)**, RH **(B)** and treatment duration **(C)** on light energy absorption, capture, and transferenergy of chrysanthemum leaves. ABS/CSm means absorption flux per cross section. TRo/CSm means trapped energy flux per cross section. ETo/CSm means electron transport flux per cross section. DIo/CSm means non-photochemical quenching per cross section. T, RH and L represent temperature, relative humidity and treatment duration, respectively.

As seen in **Figure 7A**, ABS/CSm, TRo/CSm and ETo/CSm were higher in CK treatment than that of all treatments, while DIo/CSm was lowest. With temperature increasing, ABS/CSm, TRo/CSm and ETo/CSm decreased and reached the lowest in T_41°C_, which were 20.69%, 31.87% and 49.37% lower than that of CK treatment. While DIo/CSm increased with temperature increasing, the highest DIo/CSm was observed in T_41°C_, which was 23.53% higher than that of CK treatment. As seen in **Figure 7B**, ABS/CSm, TRo/CSm, ETo/CSm, and DIo/CSm in RH_70%_ were close to that of CK treatment. ABS/CSm, TRo/CSm and ETo/CSm in RH_50%_ and RH_50%_ treatments were significantly lower than that of CK treatment, while DIo/CSm was higher. As seen in **Figure 7C**, With treatment duration increasing, ABS/CSm, TRo/CSm and ETo/CSm decreased and reached the lowest in L_9d_, which were 19.56%, 30.15% and 48.69% lower than that of CK treatment. While DIo/CSm increased with treatment duration increasing, the highest DIo/CSm was observed in L_9d_, which was 22.29% higher than that of CK treatment.

**Figure 8:**
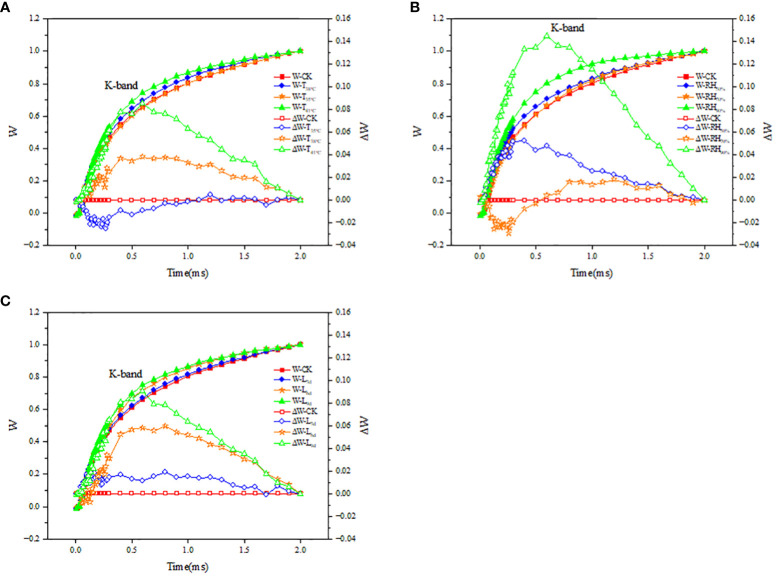
Effect of different temperature **(A)**, RH **(B)** and treatment duration **(C)** on the fluorescence differential kinetics of OJ phase of chrysanthemum leaves. T, RH and L represent temperature, relative humidity and treatment duration, respectively.

The fluorescence difference kinetics ΔW_OJ_ of the different treated OJ phases reveal their respective K-bands. As seen in **Figure 8A**, the ΔW_OJ_ curves in T_38°C_ and T_41°C_ followed the same trend and both showed a K-band, while ΔW_OJ_ curves in T_35°C_ did not show a clear K-band. As seen in **Figure 8B**, the ΔW_OJ_ curves in RH_50%_ and RH_90%_ followed the same trend and both showed a K-band, while ΔW_OJ_ curves in RH_70%_ did not show a clear K-band. As seen in **Figure 8C**, the ΔW_OJ_ curves in L_6d_ and L_9d_ followed the same trend and both showed a K-band, while ΔW_OJ_ curves in L_3d_ did not show a clear K-band.

## 4 Discussion

The light response curves parameters are important indicators that reflect the light energy utilization capacity and efficiency of plants under different environmental conditions. High temperatures affect the photosynthetic properties of plants (Li and Li, 2009). This study showed that values of AQE and P_n-max_ decreased significantly with increasing temperature, while the value of R_d_ increased. This indicated that the respiratory consumption of chrysanthemum leaves was high and photosynthetic activity was low under high temperature environments, which is in accordance with the study of Su et al. (Su and Liu, 2005) RH also affects plant photosynthesis. For example, 60% RH is the optimal level for cabbage to grow under high temperature environments, while at 90% RH under high temperature environments, cabbage was damaged and P_n_ was reduced (Han et al., 2019). In this study, AQE at 70% RH were higher than that at 50% and 90% RH. This suggested that adjusting the RH to 70% could alleviate the inhibition of high temperature stress on photosynthesis. The values of LSP, AQE, and P_n-max_ were lowest at 90% RH, suggesting that the inhibition of photosynthesis was greater under high RH environments. Similar findings were reported in different studies too (Shin et al., 2007). The prolongation of the stress time increases the damage to plant photosynthesis (Han et al., 2018). The values of LSP, AQE, and P_n-max_ were decreased with increasing treatment duration. This indicated that the degree of photosynthetic inhibition increased with prolonged stress.

Chlorophyll content is an important indicator to assess stress in photosynthetic organs and usually reflects chloroplast development and photosynthetic performance (Lu et al., 2019). Chlorophyll synthesis is a series of enzymatic reactions, and high temperature stress causes protein denaturation and lipid peroxidation of cell membranes, reducing the rate of chlorophyll synthesis (Liao et al., 2004). This study showed that the values of chlorophyll a, chlorophyll b, and chlorophyll (a+b) decreased with increasing temperature. It suggested that chloroplasts were damaged or the rate of chlorophyll synthesis was reduced under high temperature environments, contributing to the decrease in P_n_, which is in line with the study of Yang et al. (Yang et al., 2019).

High temperature leads to the decrease in chlorophyll content of plant leaves, firstly, because high temperature reduces the rate of chlorophyll synthesis (Li et al., 2020), and secondly, because the accumulation of reactive oxygen species at high temperature accelerates the degradation of chlorophyll (Yuan et al., 2017). Carotenoids are both photosynthetic pigments and endocytic source of antioxidants, which can absorb excess energy in chloroplast, quench reactive oxygen species and prevent membrane lipid peroxidation (Zhou et al., 2016). This study showed that the content of carotenoid decreased with increasing temperature, indicating that carotenoids were damaged by high temperature stress and their functions were damaged, which can lead to reactive oxygen accumulation. This is consistent with the findings of Lokesha (Lokesha et al., 2019). This also indicated that under high temperature environments, the presence of non-stomatal limiting factors for photosynthesis inhibition. Moreover, this study found that the value of carotenoids at 70% RH was highest under high temperatures. It indicated that 70% RH was able to maintain the carotenoid content of chrysanthemum leaves under high temperatures, thus absorbing excess energy from chloroplasts, quenching reactive oxygen species, and increasing P_n_.In this study, it was found that the values of chlorophyll a, chlorophyll b, and chlorophyll (a+b) decreased at 50% and 90% RH, compared to 70% RH. Hence, 70% RH could alleviate the damage of chloroplasts in chrysanthemum leaves by high temperature. The difference of photosynthetic pigment content between RH_50%_ and RH_90%_ was statistically non-significant. A recent study pointed out that at 46°C, lower RH amplifies the inhibition of the photosystem by high temperature (Lysenko et al., 2023). For high or low RH, further experiments are needed as to which has a greater effect on chlorophyll content.

Stomata are channels for the exchange of carbon and water between chloroplasts and the atmosphere and have an impact on plant physiology (Du et al., 2018). This study showed that the dominant factor in the decrease of P_n_ at 35°C and 38°C was stomatal limitation, which is concordance with the work of Fan et al. (Fan et al., 2010). In this environment, high temperature led to a large number of stomatal closures, reduced G_s_ and blocked CO_2_ diffusion, which contributed to the reduction of P_n_. Wu et al. (Wu et al., 2001) found that stomatal limitation caused inhibition of photosynthesis when plants were under mild heat stress, but inhibition of photosynthesis under extreme heat stress was caused by non-stomatal limitation. In this study, when the temperature increased to 41°C, the dominant factor was non-stomatal limitation. As can be seen from the discussion below, this dominant factor was high temperature led to disruption of the internal structure of PSII and inhibition of its activity. Stomatal morphological characteristics are related to their function and also influenced by VPD (Sinclair et al., 2007). Alineaeifard et al. (Aliniaeifard and van Meeteren, 2016) found that chrysanthemums exposed to low VPD (0.23 kPa) had larger stomatal sizes, wider pore diameters, and greater stomatal densities, resulting in higher G_s_ compared to chrysanthemums grown in a 1.05 kPa VPD environment. This study showed that the value of G_s_ was higher in a 2.00 kPa VPD environment (RH_70%_) than that in a 3.34kPa VPD environment (RH_50%_). Among three RH conditions, the value of P_n_ at 70% RH was the maximum, while R_d_ was the minimum, suggesting that 70% RH mitigated the inhibitory effect of high temperature on P_n_. High and low RH in high temperature environments can exacerbate the effects (Bunce, 2002). The study showed that the value of P_n_ decreased at 90% and 50% RH, indicating that high and low RH inhibit photosynthesis in high temperature environments. The dominant factor causing the decrease in P_n_ from 0d to 6d was stomatal limitation, but non-stomatal limitation from 6 d to 9 d. As can be seen from the discussion below, the dominant factor for the decrease in P_n_ from 6d to 9d was that prolonged stress led to disruption of the internal structure of PSII and inhibition of its activity.

WUE is the amount of CO_2_ assimilated per unit mass of water lost by leaf transpiration (Hatfield and Dold, 2019). Given that stomata control water balance, stomatal behavior has a significant effect on WUE (Li and Liu, 2022). This study showed that the value of T_r_ decreased at 35°C and 38°C. This was due to that stomatal limitation at 35°C and 38°C was a major factor in reducing P_n_, and the massive closure of stomata led to the decrease of T_r_. While the value of T_r_ increased at 41°C, because that non-stomatal limitation at 41°C was a major factor in reducing P_n_, and the increase of G_s_ led to the increase of T_r_. The value of WUE decreased with increasing temperature. This may be related to chrysanthemum leaf senescence under high temperature environments. Bunce et al. (Bunce, 2002) found that under excessively high RH conditions, increased VPD was caused by higher T_r_, which lead to the increase of G_s_. This study showed that VPD and the value of T_r_ were lower at 90% RH than that of other RH conditions. This mean that 90% RH was not favorable for transpiration of chrysanthemum leaves. Numerically, the value of WUE was higher at 70% RH than 50% RH, suggesting that 70% RH may be better for water utilization by chrysanthemum leaves than 50% RH. The value of T_r_ decreased at 3d and 6d, because that stomatal limitation at 3d and 6d was a major factor in reducing P_n_, and the massive closure of stomata led to the decrease of T_r_. While the value of T_r_ increased at 9d, because that non-stomatal limitation at 9d was a major factor in reducing P_n_, and the increase of G_s_ led to the increase of T_r_.

By analyzing chlorophyll fluorescence, it is possible to know the response of the plant to changes in environmental factors (Maxwell and Johnson, 2000). PS II is one of the most sensitive parts of the photosynthetic system to temperature stress and is closely linked to chlorophyll fluorescence (Murata et al., 2007). This study found that with increasing temperature, F_o_ and F_j_ increased. Photoinactivation usually leads to oxidative damage and inactivation of PSII reaction centers, which further leads to an increase in F_o_ (Liu et al., 2022). It indicated that high temperature resulted in inactivation of PSII reaction centers. Changes in cystoid membrane structure and organization may result in changes in F_m_ during many stress treatments (Baker, 2008). This study found that with increasing temperature, F_m_ decreased, suggesting that high temperature resulted in the changes in cystoid membrane structure and organization. F_v_/F_m_ is used to measure the maximum efficiency of PSII (Liu et al., 2020). A decrease in F_v_/F_m_ is often observed when plants are in a stress state, which represents impaired PSII function (Guidi et al., 2019). This study found that with increasing temperature, F_v_/F_m_ decreased, suggesting that high temperature resulted in impaired PSII function. In addition, this study found that ABS/CSm, TRo/CSm and ETo/CSm decreased, and DIo/CSm increased in chrysanthemum leaves under high temperature environments. This suggested that high temperatures may affect the structure of the PSII functional antenna, which reduces the ability to capture light (ABS/CSm), leading to a decrease in the excitation energy of the reduced Q_A_ (TRo/CSm) and its ability to be used for electron transfer (ETo/CSm), and an increase in heat dissipation (DIo/CSm). To further analyze the changes of PSII in chrysanthemum leaves, experiments were conducted to investigate the kinetics of fluorescence differences in the OJ phases of different treatments. ΔW_OJ_ can reflect the PSII functional antenna size and the activity of the manganese complex-dominated exocytosis complex. It was shown that K-bands appeared and ΔW_OJ_ > 0 at 38 °C and 41°C, indicating that the oxygen release complex of PSII in chrysanthemum leaves was inactivated under high temperature environments (Wakjera et al., 2013), the efficiency of plastoquinone Q_A_ in transferring electrons was decreased (Hermans et al., 2005), and PSII functional antenna size changed (Li et al., 2009). It also suggested that the main factor for the decrease in P_n_ at 41°C was that high temperature led to disruption of the internal structure of PSII and inhibition of its activity. For the different RH conditions, F_o_, F_j_, F_m_ and F_v_/F_m_ were close to those of CK at 70% RH, indicating that 70% RH mitigated the effect of high temperature on PSII activity. While F_o_, F_j_, and DIo/CSm increased, F_m_, F_v_/F_m_, ABS/CSm, TRo/CSm and ETo/CSm decreased at 50% and 90% RH. It was also shown that K-bands appeared and ΔW_OJ_ > 0 at 50% and 90% RH, indicating that both 50% and 90% RH inactivated the oxygen release complex of PSII, reduced the electron transfer efficiency of Q_A_ and PSII functional antenna size changed. With treatment duration increasing, F_o_, F_j_, and DIo/CSm increased, while F_m_, F_v_/F_m_, ABS/CSm, TRo/CSm and ETo/CSm decreased. It was also shown that K-bands appeared and ΔW_OJ_ > 0 at 6d and 9d. K-band at 9d was higher than that at 6d, suggesting that the inhibition of PSII activity was exacerbated with prolonged stress. It also suggested that the main factor for the decrease in P_n_ from 6d to 9d was that prolonged stress led to disruption of the internal structure of PSII and inhibition of its activity.

Under high temperature environment, photosynthesis is often suppressed before other cellular functions are compromised. And RH can influence photosynthesis differently according to environmental changes (Rodrigues et al., 2016). The results of this study showed that high temperature had a greater effect on photosynthesis in chrysanthemum seedlings than RH. This indicates that high temperature dominates the effect of photosynthesis under high temperature and high RH. Meanwhile, the study also showed that there was an interactive effect of high temperature and RH on photosynthesis of chrysanthemum leaves, and the interaction of the two had a significant effect on P_n_ (P<0.01). This indicates that changes in RH under high temperature conditions can significantly affect the photosynthetic rate. Excessive RH aggravated the inhibitory effect of high temperature on photosynthetic rate. This was mainly due to the fact that high RH led to low VPD, the closure of stomata, the reduction of T_r_, and the reduction of CO_2_ exchange between inside and outside the leaves (Carvalho et al., 2015). In addition, high RH produces leaf thermal overload. Elevated leaf temperatures exacerbate damage to leaf photosynthetic functions. Lieten et al. (Lieten, 2002) found that strawberry leaves under high RH conditions showed leaf tip burn. In this study, we found that the inhibition of photosynthesis rate at 90% RH was also significantly higher at 50% RH than at 70% RH under high temperature conditions. This was because stomatal closure affected leaf CO_2_ exchange and heat dissipation under low RH conditions (Ferrante and Mariani, 2018). At 70% RH, G_s_ was significantly greater than that at 50% and 90% RH, which was favorable to alleviate the inhibition of photosynthesis by high temperature.

## 5 Conclusion

This experiment investigated the effects of high temperature, RH and treatment duration on photosynthesis of “Shenma” chrysanthemum leaves. The results showed that heat stress above 35°C affects chlorophyll fluorescence parameters of chrysanthemum leaves, significantly reduced the content of photosynthetic pigments, and severely inhibited photosynthesis. Under high temperature environment, the decrease of P_n_ and photosynthetic pigment content at 70% RH was lower than the other two RH conditions and it reduced the damage of high temperatures to photosynthesis system. The dominant factor causing the decrease of P_n_ in leaves was stomatal limitation at 35°C,38°C, three RH conditions, 3d and 6d, but non-stomatal limitation at 41°C and 9d. There was an interaction between temperature and RH, with a significant impact on P_n_ (P<0.01). Under high temperature and RH environments, temperature is the main factor affecting photosynthesis, followed by RH. When the temperature reached or exceeded 35°C, adjusting the RH to 70% could effectively reduce the damage of high temperature stress on chrysanthemum leaves.

## Data availability statement

The original contributions presented in the study are included in the article/supplementary material. Further inquiries can be directed to the corresponding author.

## Author contributions

JZ: Conceptualization, Formal Analysis, Investigation, Writing – original draft, Writing – review & editing, Data curation. XJ: Conceptualization, Funding acquisition, Investigation, Methodology, Supervision, Writing – original draft, Writing – review & editing, Data curation. EA: Writing – review & editing. XL: Writing – review & editing. YZ: Funding acquisition, Methodology, Writing – review & editing. RL: Funding acquisition, Writing – review & editing.
